# Discussing a diagnosis of human papillomavirus oropharyngeal cancer with patients: An exploratory qualitative study of health professionals

**DOI:** 10.1002/hed.23916

**Published:** 2015-05-22

**Authors:** Rachael H. Dodd, Laura A. V. Marlow, Jo Waller

**Affiliations:** ^1^ Department of Epidemiology and Public HealthCancer Research United Kingdom Health Behaviour Research CentreLondonUnited Kingdom

**Keywords:** human papillomavirus (HPV), oropharyngeal cancer, health professionals, qualitative research, communication

## Abstract

**Background:**

The role of human papillomavirus (HPV) in oropharyngeal squamous cell cancer (SCC) has now been well established. Clinicians' experiences and challenges of talking to patients about HPV have yet to be explored.

**Methods:**

Fifteen health professionals caring for patients with oropharyngeal SCC were interviewed. Interviews were analyzed thematically.

**Results:**

Participants expressed mixed views about explaining the causal role of HPV to their patients. Normalizing HPV and emphasizing the positive prognosis associated with it were regarded as key messages to be communicated. Challenging experiences included managing couples in a consultation and patients' concerns about transmitting HPV to their partners. Some participants described limitations to their HPV knowledge and identified the need for further information and training.

**Conclusion:**

This study identified challenges experienced by health professionals working with patients with oropharyngeal SCC and highlights some key messages to convey to patients. Clinical guidance for health professionals and further information for patients about HPV‐positive oropharyngeal SCC are needed. © 2015 The Authors Head & Neck Published by Wiley Periodicals, Inc. *Head Neck* 38: 394–401, 2016

## INTRODUCTION

The etiological role of human papillomavirus (HPV) in oropharyngeal squamous cell carcinoma (SCC) has been well established through epidemiological studies.^1^, ^2^, ^3^, ^4^ High‐risk, sexually transmitted HPV types are thought to be responsible for up to 5% of cancers worldwide, including cervical, anal, penile, vaginal, vulva, and some oropharyngeal cancers.^5^ In the United Kingdom, incidence rates for HPV‐positive oropharyngeal SCC have doubled from 1 per 100,000 to 2.3 per 100,000 in just over a decade.^6^ The declining incidence of HPV‐negative oropharyngeal SCC, the main risk factors for which are tobacco and alcohol use, means that HPV‐positive oropharyngeal SCC now constitutes an increasing proportion of oropharyngeal SCCs overall.^7^ HPV‐positive oropharyngeal SCC seems to be biologically and clinically distinct from other head and neck cancers.^8^ Presence of HPV is associated with improved prognosis and there is increasing interest in de‐escalating treatment in patients with HPV‐positive disease.^9^

In addition to differences in tumor biology and clinical prognosis, the demographic profile of patients with HPV‐positive oropharyngeal SCC differs from those diagnosed with HPV‐negative oropharyngeal SCC. Patients with HPV‐positive oropharyngeal SCC are more likely to be men, white, under 50 years old, married, educated, and employed^8^, ^10^ compared with their HPV‐negative counterparts. This brings with it different treatment and rehabilitation needs, because patients tend otherwise to be in good health, without any traditional risk factors or comorbid disorders.^8^

For these reasons, HPV testing has been introduced as a clinical standard of care in oropharyngeal cancer in the United States, under the National Comprehensive Cancer Network guidelines.^11^ Testing tumors for HPV is also taking place in some United Kingdom centers, although no specific guidelines have been published. This change in clinical practice, together with the increasing prevalence of HPV‐positive oropharyngeal SCC, means that clinicians are beginning to discuss HPV with their patients with head and neck cancer.

A diagnosis of HPV‐positive oropharyngeal SCC is not only a cancer diagnosis, with all the associated psychological implications, but also conveys the information that the cancer was caused by a sexually transmitted infection. The sexually transmitted nature of HPV creates a potential challenge for health professionals with little experience of discussing sexual behavior.^12^, ^13^, ^14^ The possible implications of this shift were neatly summarized in a recent editorial in the British Dental Journal: “If talking about tobacco and alcohol habits have seemed like difficult subjects to raise, then talking about oral sex may present a further challenge.”^15^ Evidence from the cervical cancer literature suggests that general practitioners and practice nurses often lack knowledge of HPV and find the topic sensitive, awkward, and difficult to explain in a way patients can understand.^16^ From the patient's perspective, an HPV diagnosis has the potential to cause feelings of stigma and shame in addition to the anxiety and health concerns usually associated with abnormal cervical screening results.^17^ In the absence of any formal recommendations for discussing HPV test results with patients with oropharyngeal SCC, a recent review^18^ suggested that the cervical cancer literature could be used to provide a starting point.

The experiences of patients with HPV‐positive oropharyngeal SCC have begun to be explored; a qualitative study with male HPV‐positive oropharyngeal SCC survivors in New York found that some participants were concerned about infecting a partner with HPV and some had discontinued oral sex or deep kissing even with long‐term partners. Physicians were the primary source of information for all participants who wanted to know about HPV.^19^ In a small study exploring the information needs of patients with HPV‐positive oropharyngeal SCC in Texas,^20^ around half reported that their oncologist did not discuss issues related to HPV with them. Many of these patients sought information about HPV and cancer elsewhere.

It has been argued that health professionals have an ethical obligation to ensure accuracy and transparency when disclosing HPV as the cause of a patient's cancer,^21^ but, as yet, there have been no studies exploring this among health professionals themselves. We therefore carried out an exploratory qualitative interview study with clinicians treating patients with HPV‐positive oropharyngeal SCC to explore their experiences and the perceived challenges of talking to patients about HPV in this context. The purpose of the study was to map a broad range of experiences and views from professionals working with this patient group, and seek explanations for differences in experiences, in the hope that this work would inform future quantitative studies.

## MATERIAL AND METHODS

### Sample

Participants were health professionals caring for patients with HPV‐positive oropharyngeal SCC. We used purposive sampling to recruit participants from different disciplines in order to explore a range of perspectives. Participants were recruited via email from 8 research‐active hospitals in England and Wales (see Table 1) where HPV is discussed with patients. Potential participants were initially identified through existing contacts (2 surgeons and 2 oncologists) and we subsequently used snowballing. The first author also attended multidisciplinary team meetings at 2 hospitals in London to introduce the study and recruit participants. Initially, we aimed to purposively recruit 10 participants to include oncologists, surgeons, and nurses as they have the most contact with patients with HPV‐positive oropharyngeal SCC. As the study progressed, we included some additional professional groups also key to the care of patients with HPV‐positive oropharyngeal SCC, to try to maximize the range of views. No new themes emerged from the final 3 interviews, suggesting saturation had been achieved. We ceased data collection at this point and therefore no more interviews were conducted.^22^ Before the interview, each participant completed a short demographic questionnaire and provided written informed consent. The study was approved by the UCL Research Ethics Committee (reference number 4577/001).

**Table 1:** Sample characteristics.

Characteristic	No. of participants (*n* = 15)
Age [median (range)]	47 (33–59)
Sex	
Male	8
Female	7
Ethnicity	
White British	13
Other	2
Profession	
Surgeon	5
Clinical oncologist	3
Specialist nurse	4
Research nurse	1
Specialist radiographer	1
Speech and language therapist	1
Geographic area of sample, no.	
North West England	4
North East England	2
South West England	1
London	4
South East England	3
Wales	1

### Procedure

Semi‐structured interviews were carried out in May and June 2013. The interview followed a topic guide that was developed using the existing literature on patient experiences and previous work on HPV and cervical cancer. It covered the participants' professional background and experience of working with patients with head and neck cancer, and their experiences of and attitudes toward communicating with patients about HPV‐related head and neck cancer. Suggestions for facilitating communication in the future were also discussed.

Interviews took place face‐to‐face at the participant's workplace (*n* = 7) or over the telephone (*n* = 8), they lasted 20 to 40 minutes, and were digitally recorded. All interviews were conducted and transcribed verbatim by the first author (R.D.).

### Analysis

The interviews were analyzed using Framework Analysis.^23^ This approach involves the organization of data into a thematic framework that enables close inspection of the data by theme and by participant.

Rachael Dodd familiarized herself with the interviews by listening to, transcribing, and reading the transcripts, making notes on recurring themes and summarizing each interview. Themes were identified and developed into a thematic framework with subthemes under each main theme. Using the qualitative package NVivo 10, these data were summarized and organized into a matrix, where each column represented a subtheme and each row represented a participant. Laura Marlow and Jo Waller read half the transcripts each and were involved in developing the thematic framework.^24^ Any disagreements in interpretation were resolved by discussion.

## RESULTS

We interviewed 15 clinicians from a range of professional groups. There was an even mix of male and female health professionals, with most being from white British backgrounds. Clinical oncologists in the United Kingdom administer both chemotherapy and radiation. Characteristics of the sample are shown in Table 1.

### Significance of human papillomavirus in oropharyngeal squamous cell carcinoma

All participants regarded the role of HPV in oropharyngeal SCC as an important issue, describing HPV‐positive oropharyngeal SCC as a “different disease entirely” (participant 9, female, specialist radiographer), affecting younger, otherwise healthy patients. The rise in incidence was a key concern. Participants reported being able to tell which patients had HPV because of their appearance and demographic background, which differed from the patients they usually treated. As one oncologist reported: “When I first started in head and neck cancer practice, the stereotype of the head and neck cancer patient was pretty well fulfilled in that most of our patients were alcohol dependent, nicotine dependent and had developed head and neck cancer as a consequence of those two risk factors, but we have seen a change … over the last … decade where increasingly we're seeing younger, non‐smoking, non‐drinking patients who are on average 10 years younger and recognizing in that patient group that their HPV associated disease is the main risk factor for that” (participant 3, male, clinical oncologist).

The clinical implications of HPV‐positive oropharyngeal SCC were discussed, including patients living longer with the after‐effects of treatment and patient demands for expedited rehabilitation. One participant explained the impact of this: “we've got a longer period of survivorship for younger people who are still actively employed and so their functional rehabilitation becomes a bigger issue so that's going to be a bigger part of our case load” (participant 12, female, speech and language therapist).

### Attitudes to discussing human papillomavirus

Almost all participants had talked about HPV with their patients, but even those who did not have direct experience of this were able to express their opinions on the issue. Views about disclosing HPV as the cause of a patient's cancer varied, perhaps reflecting the lack of guidelines for discussing HPV. There was a range of views on the possible benefits of discussing HPV status with patients.

Participants who felt it important to discuss HPV status believed it was “helpful for the patients' psyche” (participant 4, male, surgeon) to understand the cause of their cancer. Sometimes patients had done their own research about HPV and had become “scared about it” (participant 10, female, clinical oncologist), making it important to provide them with accurate and reassuring information. A clinical oncologist described how patients would search for information about HPV on the internet, and felt that avoiding the issue in the clinic was unhelpful.

In centers running clinical trials, it was viewed as difficult not to mention HPV, because HPV status determined eligibility that “forces the issue” (participant 2, male, surgeon). One reason for discussing HPV was the positive prognosis of HPV‐oropharyngeal SCC, which participants felt had a direct impact on the patient and was seen as “one of the major bits of information they want to know” (participant 6, male, surgeon).

Participants who did not discuss HPV status with patients felt it unnecessary to mention HPV because it is “not offering a modifiable risk factor” (participant 3, male, clinical oncologist) and focusing on the cause may contribute to self‐blame for past behavior: “When it comes to HPV disease, I mean what can you tell them? … there's nothing that they need to adapt in their lifestyle which is going to make any difference to their outcome at all” (participant 7, male, surgeon).

One view was that patients were not concerned about the cause of their cancer during the diagnosis consultation, where other worries and discussions about treatment took priority: “not a single patient that I've met so far has asked me what's caused their cancer” (participant 7, male, surgeon). It was sometimes felt to be best to “leave it at that stage, to the patient and their family [to raise]” (participant 1, male, surgeon) because of the fact that it does not change the clinical management. The consultation was sometimes described as being patient‐directed and if patients did not ask about it, HPV “may just not come up” (participant 3, male, clinical oncologist). Some participants said they were increasingly raising the issue of HPV with their patients, whereas others described patient‐led consultations. Mentioning clinical trials prompted patients to ask questions, but 1 surgeon reported “less than 10% of patients coming back at a later stage to discuss the implications [of HPV] in a social context” (participant 1, male, surgeon).

Variations in attitudes toward communicating about HPV among health professionals were described, including a difference between surgeons and clinical oncologists: “I would say the oncologists talk about it much more easily and freely and openly, whereas the surgeons might mention it, but they don't go into how it's caused, the whole thing about HPV. No, I'd say oncologists are better at communicating about it” (participant 8, female, specialist nurse).

### Challenges to discussing human papillomavirus

Health professionals described 2 main concerns when talking to patients about HPV: the limitations of their own knowledge about the virus and discomfort talking about sexual health matters.

#### Knowledge

It was apparent that some of the health professionals felt they lacked knowledge to respond to some of the questions patients asked about HPV, with a specialist nurse reporting “no bottom to those questions” (participant 11, female, specialist nurse). It appeared there was some uncertainty about where to find accurate information, with consultants sometimes reported as giving different information to that printed in journals: “he [consultant] said ‘Oh it's not an epidemic’ … am I supposed to go with what he says, or am I supposed to go with what's in the journals?” (participant 11, female, specialist nurse). The issue was raised of not feeling well‐informed, while it was suggested that some questions are difficult to answer because of the limits of scientific knowledge: “they start asking questions about how I caught HPV and when I caught it and who I caught it from, how will I have caught it. And some of those questions are difficult to answer because we don't have the scientific knowledge at the moment … it's still quite confusing I think both for the doctors and for the patients” (participant 5, male, clinical oncologist).

Being honest with patients about not knowing the answers was advocated, because there is still scientific uncertainty: “one of the questions I have been asked is ‘So now the cancer's gone, if I have oral sex again, is it going to come back?’ I don't know that answer, I don't know if anybody does know that answer … if the questions are difficult like that, I tend to say there's a lot of research on the go at the moment and we don't have all the answers to the questions” (participant 13, female, research nurse).

There was some evidence of discomfort talking about HPV with patients. A specialist nurse said: “I'd feel out of my depth pretty quickly if people had been on the internet and they'd heard this and heard that” (participant 11, female, specialist nurse). However, confidence seemed to increase with experience, with an oncologist describing how she felt she could now honestly say “nobody knows the answer” (participant 10, female, clinical oncologist).

#### Talking about sexual health

Health professionals working with patients with head and neck cancer are not used to discussing sexual health and some participants were very aware of this: “You end up getting into the field of how was the virus transmitted and you say well it'll be broadly speaking through sexual contact and actually it's uncommon for patients to want that spelled out, but just occasionally I've got into a conversation between like as it were vaginal sex, oral sex, kissing and all of that. People want it spelled out in words of one syllable, but I think to be honest most head and neck consultants get pretty squeamish about that” (participant 2, male, surgeon).

Observations were raised about participants' colleagues: “I'm very lucky to work with some extremely talented surgeons, … but I think talking about HPV takes them out of their comfort zones somewhat … they are empathetic enough communicators to know what they're not good at and I think they'd know that they're straying out of their comfort zone; better not to get into it” (participant 3, male, clinical oncologist).

In addition, the issue of potential blame or “finger pointing” (participant 6, male, surgeon) in relation to sexual transmission was raised. One participant observed that “it can be particularly difficult when you have couples in a session” (participant 12, female, speech and language therapist). There were also concerns about not wanting to give the information in the “wrong manner” and worry about patients leaving the consultation blaming themselves. Not all participants reported difficulties talking about sexual matters, with the speech and language therapist being experienced in, for example, dealing with “difficult questions about … engaging in sexual practice when I have a stoma” (participant 12, female, speech and language therapist).

### Dealing with the impact of human papillomavirus on relationships

It was suggested that consultations could be influenced by the presence of the patients' partners and that some patients were more open to discussion about HPV without their partners present. For example, one couple had researched HPV before the consultation and the partner was concerned about whether HPV indicated infidelity: “Both husband and wife had done their homework, they knew about HPV, they knew he was likely to be HPV positive before the consultation started … but the main crux of this issue was that … the wife was [saying] ‘How's he got it, when did he get it.’ As far as the wife was concerned they'd been in a monogamous relationship for 15 years and she felt … this must be a sign that he'd been unfaithful and had other partners outside of the marriage … that was obviously causing some problems between the two of them” (participant 5, male, clinical oncologist).

The techniques described below (see Key messages) for normalizing HPV and emphasizing its high prevalence were used to try and diffuse the issues around past and/or present sexual activity. Clinicians often tried to help patients realize it was not their fault and that there was nothing they could have done to prevent their cancer.

Fear of transmission and self‐blame among partners were also described. In 1 case, a consultation had resulted in the couple ceasing sexual activity, which had led the clinical oncologist to re‐assess how issues of sexual transmission should be communicated: “we've been on a real learning curve with that [discussing HPV] and I know I got it wrong initially … we talked about it with a patient and in subsequent discussions with the support workers, that patient was not having sex with his wife anymore because he was worried he would infect her with HPV” (participant 3, male, clinical oncologist).

A surgeon also recognized the nature of the relationship as important when deciding how much to discuss in the consultation: “I was just slightly cautious … about discussing with partners the number of partners someone else has had … because obviously it was a newer relationship, not a sort of you know, 20 years married type one” (participant 6, male, surgeon).

The same surgeon (participant 6, male, surgeon) described the difficulty of talking to couples when each individual had different questions and concerns. In 1 case, the patient was more focused on the details of the treatment, but his wife was more concerned about outcome and survival. This demonstrates the different approach health professionals may have to take in joint consultations.

### Patient concerns and questions about human papillomavirus

Almost all participants gave examples of concerns and questions patients had expressed about HPV. Views differed among health professionals about what patients' primary concerns were. Some participants reported concerns mainly about diagnosis and treatment; with others reporting concerns and questions about HPV and transmission (see previous section). One specialist nurse reported an experience with a patient worried about transmitting HPV to his wife and re‐infecting himself if he continued to practice oral sex. This patient was also worried about his son and talked about getting him vaccinated: “I had this one guy who was HPV positive and obviously he was really worried about passing this onto his wife or being re‐infected by HPV if he continued to practice oral sex… He also was really worried about his son … because he knew that girls are being vaccinated against the HPV virus. He was worried that his genetic makeup, that he's developed a cancer by the HPV virus, that his son was going to and he was looking into getting his son vaccinated privately” (participant 15, female, specialist nurse).

Specialist nurses described how some patients “come armed with” (participant 8, female, specialist nurse) lots of questions about implications for transmission and the chances of becoming re‐infected, but also noted that others “probably wouldn't ask many questions” (participant 11, female, specialist nurse). Some nurses thought that patients felt more comfortable asking them questions than the surgeon or clinical oncologist. This was acknowledged by some surgeons who said that it was the specialist nurses who were asked follow‐up questions. Nurses and allied health professionals tended to see it as their role to be “the patients' advocate” (participant 8, female, specialist nurse), checking whether they had any questions after the consultation.

### Key messages

Several key messages about HPV were highlighted by participants, suggesting an agreement about core messages perceived to be useful to the patient. These focused on trying to minimize possible negative psychological responses to HPV and presenting the diagnosis in a way that was easy to understand and emphasized its positive implications. Recognizing the amount of information each patient could understand and tailoring communication to avoid overloading them was mentioned by participants as important. It was suggested that delivering information about HPV in a factual manner “can distance any emotive element” (participant 12, female, speech and language therapist), with the aim of “not making a big deal of it” (participant 10, female, clinical oncologist).

#### Normalizing human papillomavirus

Participants reported a range of ways they would try to normalize HPV infection and reduce its psychological impact. This included describing the high prevalence of HPV, highlighting that transmission is through normal sexual behavior, and using the context of cervical cancer and HPV vaccination.

#### High prevalence

HPV was often normalized by explaining that anyone who is sexually active will have been exposed to it: “it's just really a difference between how the body deals with it in different people” (participant 4, male, surgeon). The importance of communicating the fact that HPV is “a ubiquitous problem” (participant 5, male, clinical oncologist) was emphasized; a surgeon reported telling his patients that HPV is as common as flu, calling it “genital flu” (participant 6, male, surgeon).

#### Normal sexual behavior

Participants emphasized that HPV was caused by normal sexual behavior and was not an indication that the patient was promiscuous: “this is something which is associated with probably any sexual relationship … it's not like getting a dose of gonorrhea or chlamydia” (participant 4, male, surgeon). It was suggested that by being deliberately vague about the nature of transmission, it was possible to reassure the patient that almost anything could have caused it: “I say you can get it from kissing somebody … that may not be the most common way to get it but you could” (participant 5, male, clinical oncologist). Participants also emphasized the fact that HPV is an infection likely to have occurred a long time ago and that the patient had “not gone out and slept with an infected individual” (participant 4, male, surgeon).

#### Link with cervical cancer/human papillomavirus vaccination

Referring to HPV in the context of cervical cancer was reported to help patients understand that the same virus is involved in both cancers. Mentioning the HPV vaccination program was thought to convey to the patient that this virus “isn't something special” (participant 3, male, clinical oncologist) and 1 surgeon described how he would explain this: “I always explain that it's the same virus that's related to cervical cancer and that anyone that's sexual active will have been exposed to it, hence the vaccination program for pre‐sexually active individuals” (participant 4, male, surgeon).

#### No need to change behavior

When patients were concerned that they had “caused” their cancer, participants reassured them that there was no need to modify their behavior. Participants explained how they would tell patients with cancer related to tobacco and alcohol use to change their behavior as this was important for their prognosis, whereas for patients with HPV‐positive oropharyngeal SCC “there's no change in their behavior related to the sexual practice right now that you're advising them to take” (participant 2, male, surgeon), as this would not affect the outcome.

#### Positive prognosis

Participants tried to convey the positive prognosis of HPV‐positive oropharyngeal SCC, with 1 clinical oncologist reporting how this information is useful: “I think where knowledge of HPV status is useful to the patient, is enabling them to understand yes you've got head and neck cancer but we know that this particular head and neck cancer carries a much better prognosis than other forms of head and neck cancer” (participant 3, male, clinical oncologist).

Conveying the message about prognosis was viewed by most of the health professionals to help counter any feelings of blame and guilt among patients seeing this as “a bit of good news for them” (participant 7, male, surgeon) and was sometimes accompanied by a comparison to head and neck cancer related to smoking and other risk factors, using terms such as doing “better in the long term” (participant 4, male, surgeon) and a “better prognosis” (participant 6, male, surgeon).

### Professional development

#### Learning from experience

As health professionals saw an increasing number of HPV‐positive oropharyngeal SCC cases, they felt they had begun to learn what is relevant for patients. One clinical oncologist described how in the past he had “mentioned unnecessarily orogenital transmission and that's not actually relevant” (participant 3, male, clinical oncologist).

A transition was evident, from participants previously talking about contracting HPV through oral sexual behaviors, to now talking about most sexually active people contracting HPV. In some cases, participants reported having identified areas of discussion they avoided because of their lack of knowledge. They had since made an effort to find out more, resulting in increased confidence and more open discussions about HPV. Knowing the latest research and reading the literature was of upmost importance: “I mean for us it was finding out more information and having the knowledge to answer questions … then also just learning from experience about the types of things that people are asking, … doing your best to find out what the answer to that question is for the next person to ask. Because if one's [patient] going to ask, the next are” (participant 15, female, specialist nurse).

#### Learning from others

Regular team updates and feedback with colleagues were mentioned as useful to improve dissemination of information to the patient in the future. It was acknowledged that colleagues “think differently” (participant 1, male, surgeon) so working as a multidisciplinary team was viewed as very important. Attending conferences was also perceived as a valuable way to both increase knowledge about the area and learn alternative ways to discuss HPV‐positive oropharyngeal SCC.

There was agreement over the need to add to and provide consistent information to patients with HPV‐positive oropharyngeal SCC: “incorporating it in our … patient information … I certainly think that there will be serious room for improvement in that” (participant 14, male, specialist nurse).

It was also suggested that a leaflet and/or guidelines offering advice for health professionals would be useful. Another suggestion was learning from colleagues working in cervical cancer, as they have “done a very good job in that women with cervix cancer don't get immediately vilified for being sexually promiscuous and that's not the public conception of cervix cancer” (participant 5, male, clinical oncologist).

#### Training

Communication workshops and training were mentioned as a way of developing further skills: “we would be best off receiving some degree of training in terms of how to communicate this information to patients” (participant 7, male, surgeon). In some centers, communication workshops had already been carried out and participants from these centers felt they had benefited.

## DISCUSSION

This is the first study to explore the views and experiences of health professionals talking to patients about HPV in the context of oropharyngeal SCC. Views about discussing patients' HPV status were mixed. Some felt it was beneficial for the patient to know the cause of their cancer, others felt that as clinical management is not currently determined by HPV status, discussing HPV in consultations was not necessary. Most health professionals in this sample did talk to their patients about HPV, with discussions sometimes initiated by the health professional and sometimes by the patient.

Participants described several key messages about HPV that they felt were important to incorporate into their discussions with patients. Describing the high prevalence of HPV and its link with normal sexual behavior, and explaining HPV using the context of cervical cancer and HPV vaccination helped to normalize the infection. Discussion of oral sex specifically was deemed unhelpful, and recent United Kingdom evidence confirms that oral sex is commonplace, with the majority of people reporting oral sexual contact in the last year, and numbers rising in younger age groups.^25^ In the cervical cancer literature, the high prevalence of HPV has been an important message to convey to patients and has been shown to reduce stigma and embarrassment.^17^, ^26^ Previous literature has suggested because of a lack of research regarding the psychosocial impact of HPV on patients with oropharyngeal SCC, that it is helpful to look to the counseling messages used in the cervical cancer literature,^18^, ^27^ although the psychosocial implications for patients with HPV‐positive oropharyngeal SCC may be different. Our participants also felt it was important to explain that HPV‐positive oropharyngeal SCC tends to have a positive prognosis. Qualitative work with patients with HPV‐positive oropharyngeal SCC suggests that they are encouraged by this information,^19^ supporting this as a key message for health professionals to convey. A potential implication of explaining the good survival rates to patients with HPV‐positive oropharyngeal SCC is a resulting preference for de‐escalation of treatment, as 1 surgeon described. Ongoing clinical trials^9^ are exploring the possibility of de‐escalating treatment for HPV‐oropharyngeal SCC, and once the results of these trials are published clinical guidelines should be available. Until then, health professionals may still need to be prepared for conversations about de‐escalating treatment, especially with highly informed patients.

Qualitative work with patients with HPV‐positive oropharyngeal SCC suggests that questions about HPV are overshadowed by concerns about cancer.^19^ Some of the nurses that we interviewed described a lack of confidence answering questions about HPV largely because of their own lack of knowledge. Given that nurses are often the first point of contact for patients with questions, it is important that information and training is available to increase their knowledge and improve their confidence for these discussions. Surgeons and clinical oncologists also felt there was a general lack of knowledge about HPV and oropharyngeal SCC, but understood this was due to limited scientific knowledge and were generally confident explaining this to patients. Most of the health professionals we interviewed felt that additional training could help them improve their knowledge about HPV and communication with their patients with HPV‐positive oropharyngeal SCC, supporting previous research with dentists and dental hygienists.^12^ Some of the participants we interviewed felt that communicating with patients with HPV‐positive head and neck cancer was very different from communicating with patients whose cancer was related to tobacco and alcohol use. Patients with HPV‐positive oropharyngeal SCC would usually be given more information about the cause of their cancer and this often brought with it the need to discuss sexual behavior. These discussions have the potential to cause problems in relationships, demonstrated by some of the cases described in this study and previous findings from Baxi et al.^19^ This should therefore be something health professionals consider when planning treatment and recommending support for patients.

The findings from this study mirror those of similar studies in the cervical cancer literature.^16^ This suggests significant overlap in the concerns of health professionals from the 2 fields. Research into common questions asked by patients^28^, ^29^ and educational needs of health professionals from the cervical cancer literature^30^ could therefore be useful to head and neck cancer clinicians. It is important to consider, however, that the needs and concerns of patients with HPV‐positive oropharyngeal SCC are likely to differ from those of patients with cervical cancer because, in part, to that fact that a high proportion of patients with HPV‐positive oropharyngeal SCC are men.^6^, ^31^

We sampled a range of health professionals across England and Wales to gain perspectives on communicating about HPV‐positive oropharyngeal SCC from different disciplines. This work offers a useful starting point, which could contribute to the development of information for health professionals and potentially inform larger quantitative work with patients with HPV‐positive oropharyngeal SCC, with the ultimate goal of developing information for patients. Conducting this study using qualitative methods enabled the complexities of the consultation to be discussed; however, we acknowledge a number of limitations to the study. The health professionals in this study may have been those who are more comfortable talking about HPV, so it is possible that additional themes may have arisen in those who do not talk about HPV, as these were difficult to sample. Participants may also have personal biases, which could influence the discussion of sensitive topics, such as sexually transmitted infections. We were not able to draw comparisons between the different professional groups because of small numbers, but this could be an important avenue of future research. Patients' views were not explored in this study, so caution is also needed when interpreting the data about patients' concerns, as these are all from the perspective of the health professionals.

The demographic characteristics of patients with HPV‐positive oropharyngeal SCC present new challenges for health professionals in terms of the questions being asked, the factors important to the patients, and their rehabilitation and treatment needs. Experiences among health professionals differed, suggesting a need for clinical guidance for communication about HPV in this context to ensure that patients are receiving consistent messages. Further research is needed with patients to explore what being diagnosed with HPV‐positive oropharyngeal SCC means for them. There is a wealth of information available in the cervical cancer literature that could be usefully adapted for health professionals caring for patients with HPV‐positive oropharyngeal SCC.
